# Flexible, Fast and Accurate Sequence Alignment Profiling on GPGPU with PaSWAS

**DOI:** 10.1371/journal.pone.0122524

**Published:** 2015-04-01

**Authors:** Sven Warris, Feyruz Yalcin, Katherine J. L. Jackson, Jan Peter Nap

**Affiliations:** 1 Institute for Life Science & Technology & Hanze Research Center Energy, Hanze University of Applied Sciences Groningen, 9747 AS, Zernikeplein 11, Groningen, The Netherlands; 2 KeyGene N. V., 6708 PW, Agro Business Park 90, Wageningen, The Netherlands; 3 School of Biotechnology and Biomolecular Sciences, University of New South Wales, NSW 2052, Sydney, Australia; University of Georgia, UNITED STATES

## Abstract

**Motivation:**

To obtain large-scale sequence alignments in a fast and flexible way is an important step in the analyses of next generation sequencing data. Applications based on the Smith-Waterman (SW) algorithm are often either not fast enough, limited to dedicated tasks or not sufficiently accurate due to statistical issues. Current SW implementations that run on graphics hardware do not report the alignment details necessary for further analysis.

**Results:**

With the Parallel SW Alignment Software (PaSWAS) it is possible (a) to have easy access to the computational power of NVIDIA-based general purpose graphics processing units (GPGPUs) to perform high-speed sequence alignments, and (b) retrieve relevant information such as score, number of gaps and mismatches. The software reports multiple hits per alignment. The added value of the new SW implementation is demonstrated with two test cases: (1) tag recovery in next generation sequence data and (2) isotype assignment within an immunoglobulin 454 sequence data set. Both cases show the usability and versatility of the new parallel Smith-Waterman implementation.

## Introduction

Currently available next generation sequencing platforms [1] produce millions of short reads, from 30 bases up to several hundred bases, which are analyzed for SNPs [2], miRNAs [3] and other short sequences, or used for purposes such as whole genome (re)sequencing [4]. Fast, flexible and highly accurate alignment software is an important tool for analyzing such sequencing data. The alignment software should be able to process the large amounts of data within a limited timeframe, preferably on low cost and high-speed hardware. The software also needs to be highly accurate, giving the exact locations of mismatches, gaps, etc. Moreover, it is important that the application is flexible, so it can be used for many different purposes.

The Smith-Waterman (SW) algorithm is an exact method to perform local sequence alignments. The algorithm provides a dynamic programming approach of order O(n^2^), which makes the algorithm computationally slow [5]. BLAST [6] and related heuristic approaches [7] are used to search sequence databases as well as aligning sequences. Through seeding and other statistical methods, BLAST reduces the overall number of local alignments needed [6]. BLAST is very flexible and in most cases fast enough to perform the analyses required. For short sequences and highly accurate alignments, BLAST is however less suitable [8].

Dedicated software is used for finding single-nucleotide polymorphisms (SNPs) and other small differences between sequences. SOAP, for example, gives the user the location of SNPs using seeding and hash lookups, but is limited by the small number of SNPs allowed [9]. SOAP makes assumptions about SNP frequencies and uses statistical filters [10] which makes it, like BLAST, less accurate than a full SW alignment.

In recent years the use of graphics cards as platform for non-graphical data processing has taken off [11]. This programming platform provides ease of access to the computing power of the relatively cheap graphics processing unit (GPU). The programming language for NVIDIA GPUs is CUDA, which is an extension of C/C++. Numerous SW implementations have been presented upon the release of the first CUDA-enabled graphics cards and have shown that GPUs can deliver significant speed-ups compared to CPU implementations [12–17]. Some implementations, aimed at searching reads in large genome or protein databases, give a single location and highest score for each sequence. These implementations are, therefore, not able to indicate multiple hits and do not produce an alignment. Without the exact alignment it is for example impossible to find the exact location of a base change in a SNP. Other implementations have specific functionality such as accelerating protein BLAST [13].

GPGPU-based applications run on low cost, easily available hardware. The graphics cards fit in most standard desktop PCs as well as in high-end, high-performance servers. Compared to other dedicated hardware, the price-to-performance ratio favors GPGPU solutions. With the release of other GPGPU-based bioinformatics tools such as GPU-BLAST, the hardware can be used for other purposes, in contrast to dedicated hardware such as field-programmable gate arrays.

In this paper we present a new GPGPU-implementation of the SW algorithm that is not only fast and accurate, but which also generates detailed information about each alignment for inspection. The alignment information supplied includes the location of the hit, the number of matches, mismatches and gaps, as well as the alignment profile: the visual representation of the alignment. The implementation is dubbed Parallel Smith-Waterman Alignment Software (PaSWAS). PaSWAS can use any scoring matrix, so the application is able to align DNA, RNA or protein sequences. The implementation also allows for more than one profile per sequence alignment, which is useful when a sequence is contained in its target more than once or is split up in the target with a large segment between the parts.

To show the added value of PaSWAS, we analyzed two datasets that each presented a different scientific challenge. These examples are added to show the applicability of PaSWAS in general research settings and are not intended as benchmarks of any available software for each case. In the supporting information, we show how the results of PaSWAS compare to the BLAST-based analysis that were routinely performed at the institutes involved (S1 Text). Dataset (1): identity (ID) tag recovery from 454 sequence reads. An essential part of most high-throughput sequence analysis is sequence cleaning, i.e. the removal of adaptor sequences, tags or vector contamination prior to subsequent analysis, for example, by using clustering algorithms or genome assembly programs. ID tags are used to identify sample origins when biological samples are mixed before sequencing.

Dataset (2): isotype assignment of immunoglobulin 454 sequence data. Immunoglobulins (Igs) play a central role in the human immune response. Immunoglobulin genes are created through a series of genomic recombinations that bring together a number of smaller genes to create the functional rearranged genes. An Ig protein consists of two heavy and two light chains and is broadly divided into constant and variable regions. An immunoglobulin’s isotype is determined by the gene sequence which encodes the constant region. In human there are five isotypes; IgA, IgD, IgG, IgE and IgM.

In the data here evaluated, the isotypes IgE and IgG are of interest. The IgE isotype is a key component in type I allergic reactions [18]. The IgG isotype is mainly directed against invading pathogens and includes six polymorphic sub-types. The issue addressed here is to confidently classify sequence isotypes as either IgG or IgE. The portions of the constant regions captured with 454 sequencing reads are relatively short: 32 bases for the IgE and 76 bases for the six IgG isotypes. Assignment of isotype is required for downstream analyses of mutation spectras that explore the roles of the different Igs in immune responses. For example, it has been observed that non-allergic IgE sequences have significantly less mutations than allergic IgE and IgG’s [19].

## Materials and Methods

### Hardware and software

CUDA is a C/C++ extension created by NVIDIA. The development kit and the CUDA drivers are freely available from NVIDIA (www.nvidia.com). PaSWAS requires at least CUDA version 3.1 and GPU hardware version 1.2. The minimum requirements to run PaSWAS are a standard desktop computer or laptop with a recent, low-cost, consumer-grade NVIDIA-based graphics card.

Development and testing were done on a single GTX285 fitted into a computer with an Intel Core 2 Quad CPU running at 2.40GHz with 4GB of memory. The development machine also holds a GTX295, with two cores each a fraction slower than a GTX285, combined almost twice as fast. Further testing was also undertaken on a high performance computer with two C1060s and two Intel Xeon CPU X5650s running at 2.67GHz, 96GB memory and 7 TB storage. The processing environment utilized Condor [20], to provide a single grid with 19 GPU cores and 240 CPU nodes available in a single grid. The grid included fifteen GTX285, one GT295 and two C1060 Tesla graphics cards.

### Smith-Waterman Algorithm

The Smith-Waterman (SW) algorithm [5] is used in sequence analysis to find local alignments between two sequences. It requires a dynamic programming approach. In its naive form it is of order O(n^2^) for both computational resources and memory. For memory, this can be reduced to O(n) by storing only two rows from the alignment matrix [5]: during calculation of the alignment score only the previous row and the current row of the matrix are stored, together with the location of the highest value. When the calculations are finished, this location together with the highest value, the final alignment score, is returned. This process is used for searching through large databases when only the best hits are relevant and the alignment profile is not necessary in downstream analyses.

**Fig 1:**
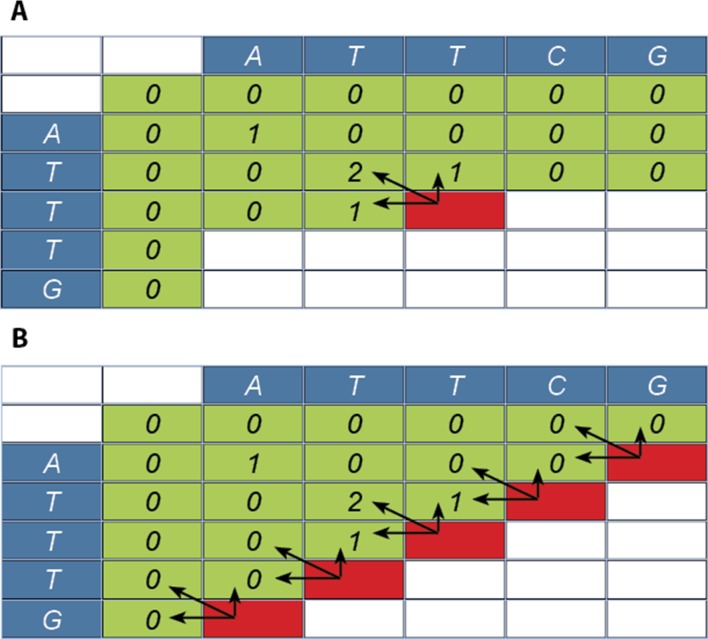
Smith-Waterman approach (A). Calculating a score for the alignment starts at the top-left and ends at the bottom-right of the matrix. The red cell is the next score that can be calculated with the arrows indicating the cells used. **Parallel Smith-Waterman approach (B).** With this algorithm the score of several cells is calculated in parallel (red cells). The arrows point to the cells used for the calculation of each new score.

For the exact local alignment an alignment matrix needs to be calculated (Fig 1A). This procedure starts in the upper-left corner and steps through the matrix left to right, top to bottom and ends bottom-right. The location of the maximum value in the matrix indicates the end of the local alignment and this alignment needs to be traced back from this cell to the start.

### Earlier parallel Smith-Waterman implementations

There are several parallel SW implementations available [12,14,15,17]. These implementations are primarily focused on searching sequence databases. They do not produce alignment profiles, which makes them fast and memory efficient but prevents visual inspection of the results. Other implementations [21,22] are capable of producing the alignment profiles, but show only one profile per alignment. These parallel SW implementations focus on two distinct properties of how the alignment matrix is filled (Fig 1B). There is a diagonal dependency of cells: cell at position (x,y) can only be processed when cells (x-1,y), (x,y-1) and (x-1,y-1) are known. In the first step only one thread is active, for cell (0,0). In each subsequent step an additional thread becomes active and halfway through the matrix the maximum number of threads are active: minimum(n,m). The number of active threads then decreases to a single thread for the last step at (n-1,m-1). This can be made more efficient by using idle threads to work on different sequence alignments [17]. In case of many sequence alignments, the processing units will be very active. For larger sequences this advantage disappears. In case the processor can calculate only a single alignment because of the length of the two sequences, there are no other cells to update and threads will remain idle. PaSWAS uses a similar approach in calculating the scores and tracebacks.

## Results

### Parallel Smith-Waterman Alignment Software (PaSWAS)

PaSWAS consists of three separate phases: (1) calculation of alignment scores, (2) determination of tracebacks and (3) production of profiles as output of the results. These steps are outlined here and the implementations are explained in more detail in the next sections. For the explanation of the application the following setup is used. On the horizontal axis there are X number of sequences, each of length N. These may be, for example reads from a sequencing platform. If a sequence is shorter than N, it is padded to length N with a special character. All sequences are placed in a single string *x* of length X*N. On the vertical axis the target sequences are placed. There are Y target sequences, each of length M. These sequences are padded when shorter than M. They are placed in a single string *y* of length Y*M.

In the first phase, the alignment matrix of each sequence alignment is calculated. The strings *x* and *y* are copied to the main (global) memory of the GPU. Each sequence alignment is calculated in parallel. Each alignment is updated over the diagonal of the matrix and starts at the top-left. At the start there will be X*Y threads active and at peak performance there are X*Y*minimum(N,M) threads active. During the entire phase the maximum value is tracked. This value is necessary to decide which tracebacks need to be calculated.

In the second phase, the traceback of each alignment is determined. Based on user-defined settings, such as the maximum value in the matrix required, the tracebacks are calculated in parallel. This implies the reverse order of the previous phase, starting at the bottom-right (Fig 1B). The profiles are stored in main memory using host page-locked memory. This keeps the data transfer between CPU and GPU to a minimum and there is no need to claim additional memory on the GPU.

The last phase is run on the CPU and consists of producing the alignment profiles. Because of the parallel nature of the algorithm, the profiles are presented in the output in random order. It is, therefore, not possible to rely on the order of the input when parsing the output. The output contains additional information about each profile, including the number of gaps, mismatches and the start and end of the alignments.

### Phase 1: calculation of alignment matrix

The GPU contains two types of memory: global memory and shared memory. Global memory is the main memory and is usually several hundred megabytes up-to 12 gigabytes in size. Shared memory is distributed across the GPU and is located physically close to the processors. It is relative small in size, but also much faster to access than global memory. Global memory is used to store the scores, the directional matrix and the strings. Because this is relatively slow memory access is therefore minimized by using the much faster shared memory on the GPU. Although shared memory can only contain 16 kilobytes per block, each of these memory blocks is shared amongst threads in the same block. For this reason, intermediate results during processing of the alignments and scores are stored in shared memory.

**Fig 2:**
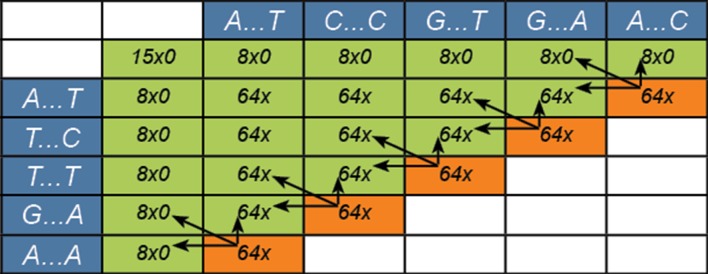
Subdivision of the alignment in PaSWAS. Each cell indicates a sequence alignment of 8 characters on the X-axis and Y-axis. The orange blocks are calculated in parallel in the same way as is depicted in Fig 1B. The arrows indicate the blocks required for the block being calculated.

Each sequence alignment is subdivided into smaller matrices of 8x8 cells (Fig 2). The occupancy calculator provided by NVIDIA (www.nvidia.com) indicates that an 8x8 block is the most efficient setting to make optimal use of the current hardware and tests confirm these settings (see S1 Fig). These 8x8 matrices map to thread blocks of 64 threads. The eight characters of the two sequences, the scores and the maximum value are stored using shared memory. This requires several data transfers from the host to global memory: the characters, the scores and maximum values from the surrounding blocks are retrieved from global memory. Without scores from a neighboring block, for example if it is the first block, scores are initialized to zero. This is all calculated in parallel.

Within each block, calculations start at the top-left and pass through the matrix via the diagonal to the bottom-right. To make use of idle threads in a block, the maximum value is determined during this pass as well. Upon completion, the resulting information is copied to global memory.

Similar to the cells within the matrix, each block depends on the three surrounding blocks. At the start, X*Y blocks of 64 threads are launched. The maximum number of thread blocks launched is (X*N*Y*M) / 64. For example, 100 reads of 400bp versus 100 sequences of 500bp will launch between 10,000 and 31,250,000 blocks at any given point in time, with each block having 64 threads. These numbers will vary between different types of graphics cards and depend on the amount of global memory available.

### Phase 2: Determination of tracebacks

After the alignment is calculated, traceback is necessary to generate the alignment profiles based on the values in the matrix.

If the maximum value within the alignment matrix complies with the requirements set by the user, it is marked as the start of the traceback. This includes a check of the value against a user-provided minimum value to give multiple profiles per alignment.

This phase is the opposite of the previous phase: the application starts at the bottom-right block and the bottom-right cell of this block. When a cell is the starting point of an alignment profile, the information about this starting point is copied to the memory of the computer (host). The x, y and score are copied, as well as the direction the score was coming from: the cell located at the left, up or upper-left. These starting points are calculated in parallel as well, which is taken into account when copying the data to the host. A procedure has been implemented to ensure that each starting point is stored without forcing the program into a sequential flow, which would otherwise slow down execution. On the host an array is allocated to store the starting points. The GPU has an integer index which points to the first available position. When a thread detects a starting point this index is increased by one using the atomic function of the hardware [23]. This function guarantees that this thread is the only process accessing this value. The host also has a matrix allocated to store the direction. This directional value is also copied directly to the host.

The thread now marks the location where the score comes from as negative in the matrix. The SW algorithm requires that all values are larger than, or equal to, zero. A negative sign is therefore appropriate to mark the traceback on the GPU without the need for additional memory space. Each thread mapped to a cell therefore checks not only for starting points, but also for negative values. If a negative value is found, the direction is copied to the host and the score at that location will be marked as negative. At the end of each block all negative values are copied to global memory for the surrounding blocks to continue the traceback until the block in the upper-left corner is reached.

### Phase 3: Production of profiles

**Fig 3:**
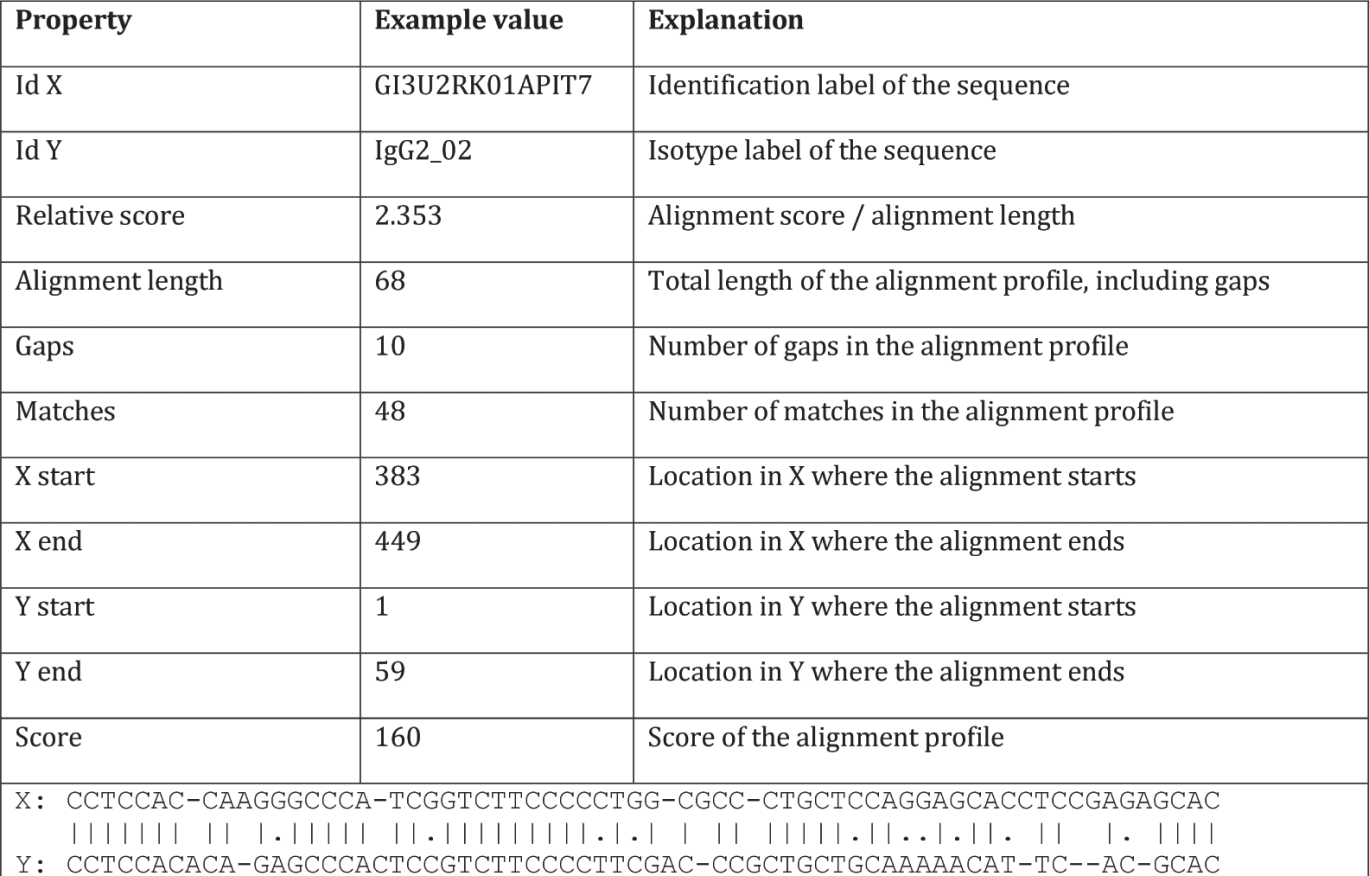
Output of PaSWAS for a single alignment. The property column gives the name of the property available, followed by an example of a value for each property. The last row shows the alignment profile of X versus Y with ‘|’ indicating a match, ‘-‘ a gap and ‘.’ a mismatch.

This phase is relatively short. The list produced in phase 2 contains the locations in the direction matrix where to start the traceback. This list is now processed. The application goes through this matrix using the directional information and counts the number of gaps, mismatches and matches and creates three strings: the sequence, the target with the gaps marked and the alignment profile. An example of the output is shown in Fig 3.

### Test case 1: Tag recovery

In the first test case, PaSWAS is used for short adaptor and tag detection and subsequent cleaning of a set of sequence reads. The data set contains 401,824 reads with lengths between 50 and 600 nucleotides from a 454 GS Titanium platform (www.roche.com), comprising 28 different samples labeled by a sequence tag that defines the origin of each read. Each read has the following structure:

[5' ID tag][5' primer sequence][genomic sequence][3' primer sequence][3' ID tag]

The 5' ID tag is five nucleotides long. The neighboring 5' primer sequence is either 19 or 21 bases long and is used for sample identification. The 3' primer sequence and 3' ID tag have the same functionality as their 5' counterparts. In this study only the results of the 5' data are used. The 3' data is only used to support the 5' analysis and is not used for primary identification.

Identification of the exact start site of the primer is necessary to retrieve the tag and the exact end of the primer is necessary to prevent contamination of the genomic sequence. During the sequencing process small numbers of errors can be introduced. A read may therefore contain gaps, nucleotide substitutions and nucleotide additions compared to the primers and tags used. Perfect sequence matching of every read is therefore unlikely. Adding or deleting parts of the genomic sequence makes detecting small changes such as SNPs in the genomic sequence more difficult.

PaSWAS was used to align the 5' primer sequences to the reads and the resulting alignments were used to get the tag immediately adjacent to this primer sequence.

Including the reverse complement of the primer sequence, this resulted in 1,607,296 sequence alignments. PaSWAS ran for 92 seconds, calculating 17,470 alignments per second (roughly 1.2 giga cell updates per second). In the 401,824 reads 357,395 5' ID tags were identified by PaSWAS (Table 1).

**Table 1:** ID tag recovery in a 454 data set.

**Data**	**PaSWAS results**
Number of reads processed	401,824
Number of primers used	2
Time (sec)	92
Recovery 5’ ID tags	357,395 (88.9% of reads)

Results of ID tag recovery in a 454 data set obtained with PaSWAS.

The performance of PaSWAS (Table 2) is based on a relative score. This score is defined as the alignment score divided by the alignment length. The match score is set to 5.0, so a relative score of 5.0 indicates a perfect match over the entire alignment. In this data set, 44.9% of reported alignments contain the entire primer sequence with no mismatches and/or gaps. There are 53.5% hits with a relative score of 5.0. 8.6% of the hits have mismatches/gaps at the start or end of the primer and a full match over the local alignment (53.5–44.9). A substantial number of alignments have a less-than-perfect match, which shows that detecting only perfect matches over the entire length of the primer will result in substantial data loss.

**Table 2:** Performance of PaSWAS in ID tag recovery.

**Description**	**Relative score (score / length)**	**Hits (%)**
Full primer recovery	X = 5.0	44.9
Perfect alignments	X = 5.0	53.5
Some gaps / mismatches	4.0 ≤X ≤ 5.0	35.3
Low quality alignments	3.0 ≤ X ≤ 4.0	10.5
Very low quality alignments	X ≤ 3.0	0.6

44.9% of the hits contains the full primer sequence. PaSWAS presents an accurate alignment tool able to retrieve degenerated tags. The relative score used is defined as the alignment score divided by the alignment length.

**Fig 4:**
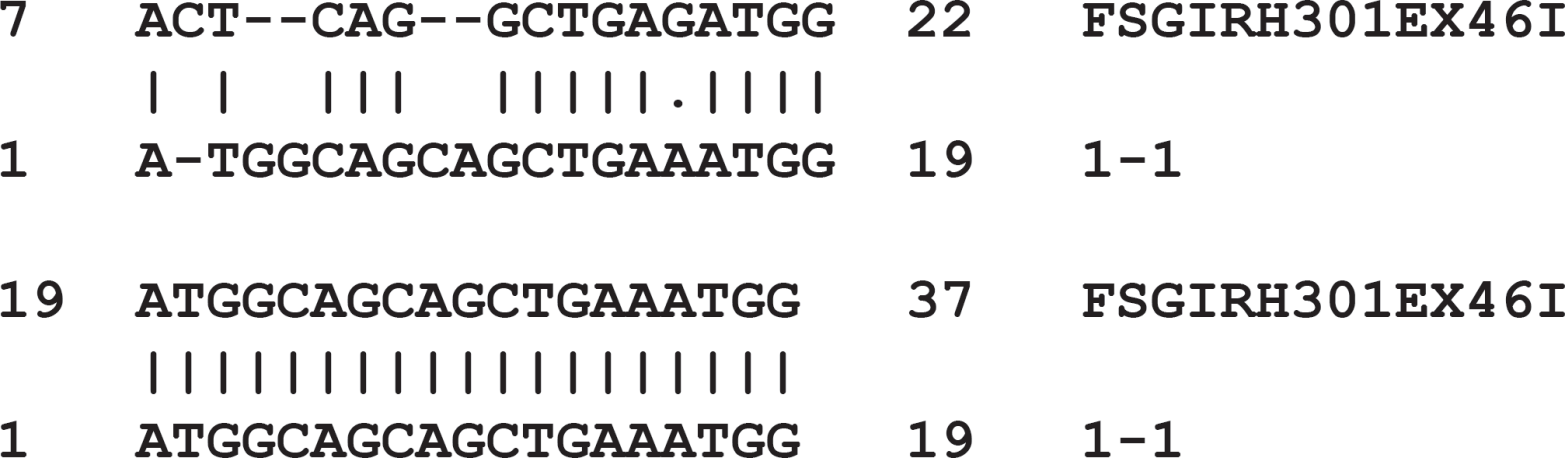
Example sequence alignment with multiple hits per alignment. In this figure the alignment of a primer sequence (1–1) to a 454 read (FSGIRH301EX46I) is shown. The top profile shows the location of degenerated primer. The bottom profile shows the best alignment, starting at position 19 in the read.

A possible source of errors is a primer that matches at more places, either because of sequencing errors or by chance. PaSWAS is the only GPGPU-based SW implementation now available which allows to investigate such cases. PaSWAS is able to produce multiple hits per sequence alignment and is therefore capable of detecting the correct location of the primer. Fig 4 shows the location of the primer in the top alignment profile and the best hit, starting at location 19 in the read, in the bottom alignment. In this case the second-best hit allows for proper tag detection in this read.

The results presented for this case show that the accurate alignment accomplished by PaSWAS has considerably added value for retrieving degenerated tags.

### Test case 2: Isotype assignment of immunoglobulin genes

To show the flexibility of PaSWAS with different data types, we used an immunoglobulin data set. Given short (32–76 bases) sequences of immunoglobulin constant regions, the aim is to classify the correct isotype of the immunoglobulins for downstream analysis of the spectra of mutations.

The 454 data set consisted of 55,295 reads with candidate IgE and IgG sequences. Of these, the IgE isotype classification required confirmation. This confirmation was based on comparison with the known IgE and IgG sequences.

On a GTX285, it took PaSWAS 154 seconds to perform the 774,130 alignments (including reverse complement), representing a rate of 5,026 alignments per second or 72 mega cell updates per second.

**Fig 5:**
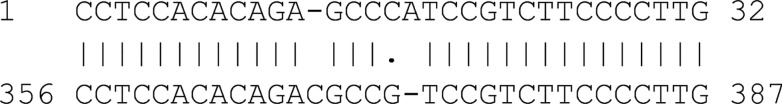
Alignment generated by PaSWAS. The top entry is part of the sequence of the IgE constant region and the bottom entry is part of a 454 read. A dot indicates a mismatch in the alignment and a minus symbol indicates a gap. Mismatches are clearly distinguishable in the profile, with two gaps and one mismatch.

PaSWAS identified 32,947 IgE sequences and 17,505 IgG sequences in the data set (Table 3). As a consequence, there are 4,843 sequences for which the isotype could not be confirmed. For the sequences classified, the number of unique sequences and the number of mutations compared to the sequences of the known isotypes (IgE or IgG) was determined with PaSWAS. In Table 4, the number of mutations and unique sequences identified are presented. For the IgE sequences, 89,895 mutations became available for analysis and for the IgG sequences 115,335 mutations. Fig 5 shows an example of the alignment profile generated by PaSWAS that is essential for such mutational analyses.

**Table 3:** Classification of immunoglobulin sequences by PaSWAS.

**Immunoglobulin classification**				
	IgE	IgG	Unclassified	Total
Classified by PaSWAS	32,947	17,505	4,843	55,295

The table shows the number of sequences classified by PaSWAS as either IgE or IgG. A small subset of the dataset (11.4%) could not be classified as either IgE or IgG.

**Table 4:** Number of mutations found in the classified immunoglobulin IgE and IgG isotype data.

	Identified by PaSWAS
**IgE**	
Total mutations	89895
Total unique sequences	7120
**IgG**	
Total mutations	115335
Total unique sequences	6109

For both the isotypes IgE and IgG the total number of mutations and number of unique sequences identified with PaSWAS is given.

Both cases presented show the applicability of PaSWAS. It is able to handle real-life sized data sets fast enough while delivering the accuracy of a full SW.

## Discussion

In this paper we present PaSWAS as novel parallel implementation of the SW algorithm on a GPU. There are several advantages of PaSWAS over other SW implementations. The first advantage is that PaSWAS produces complete alignment profiles with gaps, mismatches and matches and is not limited to one hit per sequence alignment. To our knowledge, the parallel SW implementations currently put forward in the literature do not allow inspection of the actual alignments and do not present multiple hits per alignment. PaSWAS can, if so desired, deliver a virtually unlimited number of local alignments for a single sequence comparison (Fig 4). This is essential for comparing short sequences (reads) to long sequences such as genomic regions.

PaSWAS is not limited to particular types or lengths of sequences. It can handle RNA and protein sequences just as easily as DNA sequences and allows the use of different scoring matrices. Moreover, PaSWAS is relatively fast.

Both test cases presented show that the innate accuracy of the SW algorithm as implemented in PaSWAS can give added value such as detailed alignment information necessary for SNP or degenerated tag detection. The example only shows the practical applicability of PaSWAS for this type of application. Other software such as Reaper [24] or Trimmomatic [25] may be as suitable or better than PaSWAS for tag detection. Proper benchmarking would require more and more detailed comparisons of different software. This is considered a challenge for the future.

The current output of sequencing technology combined with the speed of PaSWAS implies that thousands to millions of sequences can be recovered and/or identified for further analysis, resulting in efficient use of sequence data and effective analysis.

PaSWAS is accurate and fast, but for large genome-wide analysis, for example analyses of millions of sequence reads of the human genome, a cluster of GPUs is still required to make computations feasible in time. Each year the number of floating point operations per second performed by a GPU is increasing rapidly, so such genome-wide analyses will become feasible in the near future.

The added value of PaSWAS is particularly in producing alignment profiles. These profiles can be inspected visually or automatically for gaps and mismatches to allow, for example, SNP detection. To allow for multiple hits per sequence alignment and additional profile information, PaSWAS requires additional storage and significantly more calculations compared to searching through a database. It is therefore inappropriate to compare the speed of this algorithm with search-only GPU-applications. PaSWAS is slower due to the additional calculations and memory access. PaSWAS currently focuses on local alignments. Features to be included in the future are gap extension penalties, codon insertion/deletion scoring and a user-friendly framework so the application can be easily plugged into existing analysis pipelines. This is likely to result in different versions of the software, because we expect that some of these features will present a performance penalty. Such a penalty may not be attractive for all uses or users.

Other features will be faster than calculating local alignments, because they will require significantly less administration and calculations.

A revised version of PaSWAS is under development. The algorithm and the CUDA code will remain the same, but the data processing, logging, output formatting etc. will be written in Python. This application will take care of the compiling of the CUDA code, will have an user-friendly command line interface and new functionalities such as the possibility of trimming sequences. It will give developers easier opportunities to add new functionalities or incorporate the PaSWAS code in their own applications. One the other key will be the ability to output SAM files. We intent to publish this Python version of PaSWAS in due course.

The current implementation is based on the vender-specific CUDA platform. This limits the use of PaSWAS to NVIDIA-based graphics cards. These cards are widely available but to make PaSWAS run on other brands of GPUs, on different types of CPUs and on other many-core architectures, we are currently working on an OpenCL (https://www.khronos.org/opencl/) implementation of PaSWAS.

With such developments, the use of the SW algorithm on GPUs and CPUs will continue to present even more attractive approaches for the analyses of (next generation) sequence data.

## Supporting Information

S1 Text Comparison of PaSWAS to BLAST-based approaches. This document shows how the results of PaSWAS for the two test cases compare to the BLAST-based analysis that were routinely performed at the institutes involved.
(DOC)

S1 Fig Timing per block size. This plot shows the speed of the PaSWAS algorithm (y-axis) for different thread block sizes (x-axis).
(TIF)
