# Supplementary material for: Flexible, Fast and Accurate Sequence Alignment Profiling on GPGPU with PaSWAS
Source: PLoS One. 2015 Apr 1;10(4):e0122524. doi: 10.1371/journal.pone.0122524 (PMC4382095; doi:10.1371/journal.pone.0122524)
Supplement: S1 Text — This document shows how the results of PaSWAS for the two test cases compare to the BLAST-based analysis that were routinely performed at the institutes involved. (DOC) [file pone.0122524.s001.doc]

# Comparison of PaSWAS to BLAST-based approaches

To show the value of PaSWAS relative to ongoing procedures in laboratories, we here show the comparison of the results from PaSWAS with the results from routinely used BLAST-based approaches. The Blast-based analyses were used as standard in the labs we collaborated with. Such standard analysis should be considered as matter of fact; it may not be of have been the best possible solution for such analyses. Many more options for software are available. However, often biology-oriented laboratories stick to known procedures and do not go or dare to go into benchmarking or implementing new software. Although we show that PaSWAS outperforms these standards, the comparison here shown is not meant as benchmark of any available alternative software. As the comparison is not meant as or suitable as benchmark, default BLAST settings were used and the BLAST was not necessarily optimized for this particular case .The results only demonstrate that PaSWAS is suitable for such analyzes and PaSWAS out-performs the methods used routinely.

# Tag recovery

PaSWAS is used for short adaptor and tag detection and subsequent cleaning of 454 sequence reads. The results are compared to the output and performance of a hardware-accelerated (FPGA) platform. This platform consists of dedicated hardware running a BLAST-like algorithm setup with default settings and is routinely used but not optimized for this particular case. Filtering of the data was performed after the BLAST analysis. Details of the data are given in the main text of the paper.

The number and identity of tags retrieved by PaSWAS were compared to the results of the hardware platform analysis. The results are given in Table 1. PaSWAS ran for 92 seconds, calculating 17,470 alignments per second (roughly 1.2 giga cell updates per second). In the 401,824 reads 357,395 5' ID tags were identified by PaSWAS, compared to 287,995 by the BLAST-based hardware platform. These 287,995 tags are for 99.7% contained in the set of the ID tags identified by PaSWAS. Hence only 0.3% of the tags identified by the hardware platform are not identified by PaSWAS. Compared to the standard set-up, PaSWAS retrieves 19.5% more tags, involving 88,9% of the reads. As a consequence, considerably more useful information is extracted from the sequence data set.

# Isotype assignment of immunoglobulin genes

Aim is to classify the correct isotype of the immunoglobulins for downstream analysis of the spectra of mutations based on the 32-76 bases short sequences of the Ig constant regions.

The result of the BLAST analysis used in the laboratory is compared to the results of a PaSWAS analysis. Details of the data are given in the main paper.

The 454 data set consisted of 55,295 reads with candidate IgE and IgG sequences. Of these, the IgE isotype classification required confirmation. This confirmation was based on comparison with the known IgE and IgG sequences.

On a GTX285, it took 154 seconds to perform the 774,130 alignments (including reverse complement), representing a rate of 5,026 alignments per second or 72 mega cell updates per second. The BLAST analysis used for comparison was part of a larger grid application, that ran for several hours on a multicore grid. The pre- and post-processing was part of the analysis on the grid implementation used. The results are shown in Table 2.

PaSWAS identified 15.4% more IgE sequences and 5.3% more IgG sequences in the data set compared to the BLAST approach. As a consequence, there are 52.1% fewer sequences for which the isotype could not be confirmed. For the sequences classified, the number of unique sequences and the number of mutations compared to the sequences of the known isotypes (IgE or IgG) was also compared between PaSWAS and BLAST (Table 3). For the IgE sequences, PaSWAS identified 8,906 (11.0%) more mutations for analysis and for the IgG sequences 3,001 (2.7%) more than the BLAST approach .Both laboratories were pleased with the way PaSWAS extracted more useful data from the data than the methods they were used to. For proper benchmarking, however, it will be interesting and necessary to compare the results of PaSWAS with the results of other dedicated software and validation in the lab.

|  | **PaSWAS** | **Hardware platform** |
| --- | --- | --- |
| Number of reads | 401,824 | |
| Number of primers | 2 | |
| Time (sec) | 92 | 120 |
| Recovery 5’ ID tags | 357,395 (88.9% of reads) | 287,995 (71.7% of reads) |
| **Overlap** | **99.7%** | |
| **Relative performance** | **119.5%** | |

**Table 1. ID tag recovery in a 454 data set.** Comparison of the results of ID tag recovery in a 454 data set obtained with PaSWAS and the hardware platform.

| **Immunoglobulin classification** | | | | |
| --- | --- | --- | --- | --- |
|  | IgE | IgG | Unclassified | Total |
| PaSWAS | 32,947 | 17,505 | 4,843 | 55,295 |
| BLAST | 28,549 | 16,628 | 10,118 | 55,295 |
| % change | **+15.4** | **+5.3** | **-52.1** |  |

**Table 2. Classification of immunoglobulin sequences by PaSWAS and BLAST.** The percentage change indicates the difference in number of sequences classified by PaSWAS compared to the BLAST-based approach.

| **IgE** |  |  |  |  |
| --- | --- | --- | --- | --- |
|  | PaSWAS | BLAST | Difference | Difference (%) |
| Total mutations | 89895 | 80989 | 8906 | 11.0 |
| Total unique sequences | 7120 | 6387 | 733 | 11.5 |
| **IgG** | | | | |
| Total mutations | 115335 | 112334 | 3001 | 2.7 |
| Total unique sequences | 6109 | 5917 | 192 | 2.0 |

**Table 3: Number of mutations found in the classified immunoglobulin IgE and IgG isotype data.** For both the isotypes IgE and IgG the total number of mutations and number of unique sequences identified with either PaSWAS or BLAST is given**.**
