# Supplementary figures and images for: Flexible, Fast and Accurate Sequence Alignment Profiling on GPGPU with PaSWAS
Source: PLoS One. 2015 Apr 1;10(4):e0122524. doi: 10.1371/journal.pone.0122524 (PMC4382095; doi:10.1371/journal.pone.0122524)

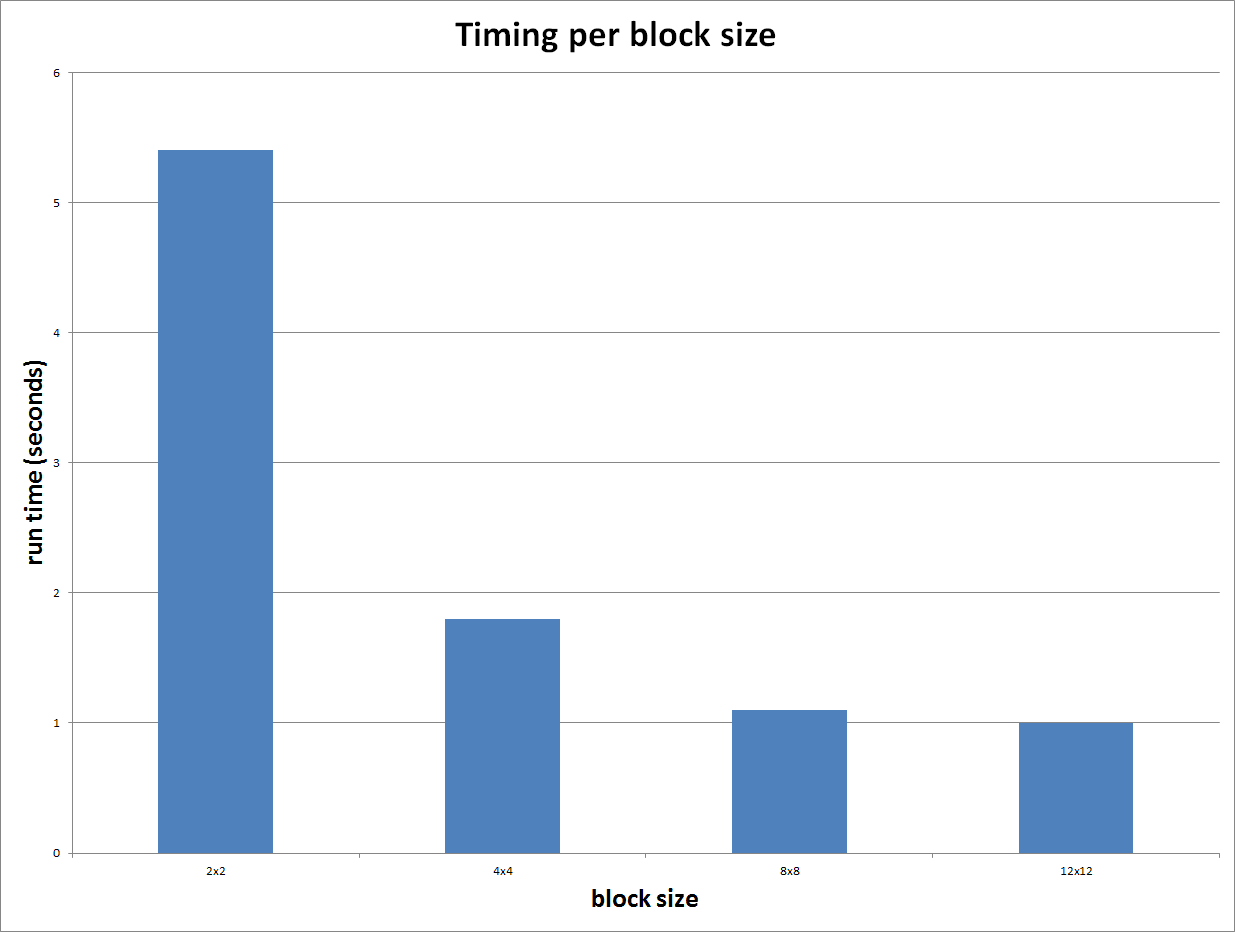

Supplement: S1 Fig — This plot shows the speed of the PaSWAS algorithm (y-axis) for different thread block sizes (x-axis). (TIF) [file pone.0122524.s002.tif]
